# The role of B7-H4 in ovarian cancer immunotherapy: current status, challenges, and perspectives

**DOI:** 10.3389/fimmu.2024.1426050

**Published:** 2024-08-29

**Authors:** Lu Zhou, Yuanqiong Duan, Kaiyu Fu, Mengpei Zhang, Kemin Li, Rutie Yin

**Affiliations:** ^1^ Department of Obstetrics and Gynecology, West China Second University Hospital, Sichuan University, Chengdu, China; ^2^ Key Laboratory of Birth Defects and Related Diseases of Women and Children (Sichuan University), Ministry of Education, West China Second University Hospital, Sichuan University, Chengdu, China

**Keywords:** B7-H4, ovarian cancer, tumor microenvironment, immunotherapy, immune checkpoint inhibitors

## Abstract

Immunotherapy stands as a critical and auspicious therapeutic approach in the fight against cancer nowadays. Immune checkpoint inhibitors, in particular, have garnered widespread employment and delivered groundbreaking therapeutic outcomes across various malignancies. However, the efficacy is unsatisfactory in the ovarian cancer. The pressing concerns of the substantial non-response rate require immediate attention. The pursuit of novel targets and the formulation of synergistic combination therapy approaches are imperative for addressing this challenge. B7-H4, a member of the B7 family of co-inhibitory molecules, exhibits high expression levels in ovarian cancer, correlating closely with tumor progression, drug resistance, and unfavorable prognosis. B7-H4 has the potential to serve as a valuable biomarker for evaluating the immune response of patients. Recent investigations and preclinical trials focusing on B7-H4 in the context of ovarian cancer immunotherapy highlight its emergence as a promising immunotherapeutic target. This review aims to discuss these findings and anticipate the future prospects of leveraging B7-H4 in ovarian cancer immunotherapy and targeted therapy.

## 1 Introduction

According to the latest global cancer statistics for 2022, it is estimated that there will be over 320,000 new cases of ovarian cancer (OC) worldwide, with approximately 200,000 associated deaths (1). Both the incidence and mortality rates of ovarian cancer (OC) are increasing, with a trend towards affecting younger individuals (2–4). Early symptoms in OC patients often manifest subtly, leading to a low diagnosis rate, and consequently, a prevalence of advanced and metastatic cases. This scenario imposes a significant healthcare burden on women.

In the current treatment approach for OC, an initial evaluation is essential. For patients without contraindications for surgery and where satisfactory Primary cytoreductive surgery (PCS) can be achieved, comprehensive staging surgery should be performed. For patients unable to undergo satisfactory debulking surgery, neoadjuvant chemotherapy followed by interval cytoreductive surgery (ICS) is recommended. The majority of patients require adjuvant chemotherapy postoperatively, with paclitaxel and carboplatin being the standard first-line chemotherapy regimen for advanced ovarian cancer. Depending on the stage and pathological subtype, the addition of bevacizumab to the TC regimen may be considered. Maintenance therapy with PARP inhibitors and/or bevacizumab should be selected based on BRCA and homologous recombination deficiency (HRD) gene test results. Nevertheless, a considerable proportion of women experience relapse, develop drug resistance and ultimately die of the disease.

Over the past two decades, immunotherapy has undergone rapid advancement and reshaped the management landscape of numerous cancers. The most extensively studied and commonly employed immunotherapy for solid tumor is immune checkpoint inhibitor (ICI) therapy, including anti-programmed death-1 (PD-1), anti-PD-L1, and anti-cytotoxic T lymphocyte-associated antigen-4 (CTLA-4), which has demonstrated efficacy in multiple clinical settings. Mounting evidence suggests that OC possesses the capacity to elicit endogenous anti-tumor immune responses, thereby indicating the potential benefits of immunotherapy for patients. Clinical trials reports indicated that the objective response rates to ICI in patients with recurrent or refractory ovarian cancer range from 6% to 15%, with some studies reporting rates as high as 45% (5–8), suggesting an already encouraging outcome. Currently, immunotherapy is recommended by the NCCN guidelines for the treatment of recurrent epithelial ovarian cancer, fallopian tube cancer, and primary peritoneal cancer with MSI-H/dMMR or TMB-H status, including patients sensitive or resistant to platinum-based therapy. Moreover, some combination therapy with ICI exhibits higher response rates compared to monotherapy (5). However, challenges such as low response rates and recurrence persist. Therefore, further investigation into the mechanisms underlying non-response, identification of precise and appropriate targets, and exploration of safer and more effective combination therapy regimens is imperative.

Research increasingly highlights the role of immunosuppression in the development and prognosis of OC. The tumor microenvironment (TME), a complex structure around tumor cells, includes blood vessels, immune cells, fibroblasts, adipocytes, and the extracellular matrix (9, 10). Tumors employ various suppressive mechanisms within the TME to evade host immune surveillance actively (11). Most solid tumors, including OC, harbor infiltrates of immune cells from myeloid and lymphoid lineages, orchestrating the construction of the TME during tumor progression, a phenomenon referred to as Tumor Infiltrating Lymphocytes (TILs) (12). TILs encompass a spectrum of immune cell types, including CD4+ and CD8+ T cells, B lymphocytes, Natural Killer (NK) cells, macrophages, and dendritic cells (DCs) (12, 13). While some TILs eliminate tumor cells, others, like regulatory T cells (Tregs), suppress immune responses, aiding tumor evasion. TILs enhance the expression of interferon-gamma(IFN), interleukin-2(IL-2), and lymphocyte-attracting chemokines in OC, associating with a good prognosis in patients with OC (14–18).

B7-H4, a transmembrane glycoprotein belonging to the B7 family of co-inhibitory molecules, exhibits widespread expression in various malignancies, particularly OC (19, 20). It is implicated in multiple processes including tumor development, immune evasion, and immune resistance (21–23). Several monoclonal antibodies, bispecific antibodies, and antibody-drug conjugates (ADCs) targeting B7-H4 have progressed to preclinical or clinical trials, demonstrating promising initial results. This review consolidates the research advancements concerning B7-H4 in OC immunotherapy and presents future perspectives.

## 2 B7 family and B7-H4

B7-H4, also called B7x、B7h.5、B7-Homolog 4 (B7-H4、v-set domain containing T cell activation inhibitor 1 (VTCN1), or B7 superfamily member 1 (B7S1), was first identified in 2003 (24, 25). It belongs to the type I transmembrane glycoprotein class and is a constituent of the B7/CD28 superfamily, situated on chromosome 1p12/13.1. Encoded by the VTCN1 gene, B7-H4 negatively regulates T-cell function (26–29). The B7 family plays a pivotal role in modulating the immune response, preventing excessive activation. The B7 family, which includes co-stimulatory and co-inhibitory molecules, is one of the most crucial second-signaling pathways in T-signaling activation. B7-H4 emerges as a novel member of the B7 family of co-inhibitory molecules, functioning as an immune checkpoint modulator involved in regulating anti-inflammatory and immune responses. Structurally, B7-H4 comprises two immunoglobulin (Ig)-like domains and a large hydrophobic trans-membrane domain followed by two intracellular amino acids. It shares variable levels of amino acid sequence identity with other B7 family members: B7-1 (12%), B7-2 (13%), PD-L1 (18%), PD-L2 (18%), and B7-H3 (24%) (30). B7-H4 generally expresses on antigen-presenting cells (APCs) and is induced by local cytokine production such as Il-6, IL-10, and hypoxia (31, 32). The receptor for B7-H4, distinct from other members of the CD28 receptor family, is expressed on activated T cells (30), suggesting a potential role for B7-H4 in the regulation of T-cell activation and exhaustion.

B7-H4 exhibits widespread expression across various malignancies and is inversely associated with patient prognosis and T cell infiltration within tumors. Moreover, it is implicated in potential associations with drug resistance (31, 33–35). Currently, the role of B7-H4 in tumor immunity has been partially elucidated in solid tumors such as liver cancer, breast cancer and OC, yet further research is needed to fully uncover its complete and potential mechanisms. Additionally, there may be more than one receptor for B7-H4 on the surface of T cells, and the precise molecular structures of these receptors remain undetermined.

## 3 Expression and clinical significance of B7-H4 in OC

B7-H4 expression is notably minimal, if not entirely absent, in normal ovarian tissue as well as other healthy tissues such as lung, liver, pancreas, spleen, thymus, and kidney (36). Nonetheless, elevated levels of B7-H4 mRNA have been detected in these tissues, indicating potential translational or post-transcriptional regulatory mechanisms governing its expression. Of note, B7-H4 exhibits significant upregulation in tumor tissues of OC patients (19–21, 37–40), a trend widely associated with advanced cancer stage and heightened aggressiveness according to the majority of studies. This suggests that B7-H4 exhibits a distinct expression pattern compared to PD-L1 in human cancers, characterized by heightened sensitivity and specificity. B7-H4 predominantly localizes on the membrane surface of antigen-presenting cells (APCs) and both intracellular and membrane surfaces of OC cells. Notably, tumor-associated macrophages (TAMs) expressing membrane-bound B7-H4 rather than tumor cells, exert an inhibitory influence on T cell immunity (27, 31, 41, 42), suggesting the importance of elucidating the mechanism and functional implications of B7-H4’s subcellular localization in OC. In OC cells, TAMs induce regulatory T cells (Tregs) to secrete IL-6 and IL-10, thereby promoting B7-H4 expression on APCs. B7-H4 overexpression has been documented in 48%, 55%, and 67% of patients at stages I, II, and advanced stages of OC, respectively (37). Additionally, B7-H4 is highly expressed in primary and metastatic serous, endometrioid, clear cell, and epithelial ovarian cancers, while its expression is lower in mucinous and non-epithelial ovarian cancers (37, 43–45). Zang et al. collected tumor tissue samples from 103 patients with epithelial ovarian cancer and constructed a tissue microarray, demonstrated 100% expression of B7-H4 in ovarian junction tumors and OC. While the intensity of B7-H4 expression varied among different pathological subtypes, these differences did not reach statistical significance (21, 46). Further studies incorporating a larger sample size are needed for more comprehensive investigation. Recent studies have consistently demonstrated that B7-H4 is upregulated in 92% of high-grade serous ovarian carcinoma (HGSOC) cases at diagnosis (n = 12) and maintains stable or increased expression following standard-of-care chemotherapy. Moreover, B7-H4 remains consistently overexpressed or more highly expressed across metastatic sites even after the development of multidrug resistance (47).

A soluble form of B7-H4 exists, and soluble B7-H4(sB7-H4) protein can be detected in the serum or plasma of OC patients. Simon et al. showed that the sensitivity of OC detection increases from 52% for CA-125 alone to 65% with 97% specificity when used in combination with B7-H4. Simon et al. also demonstrated that serum B7-H4 levels are more stable compared to CA-125 and do not fluctuate in patients with inflammatory diseases or during pregnancy (45, 48). Gyllensten et al. employed high-precision proteomics to identify 1,463 plasma proteins and validated their findings using two cohorts of previously untreated patients with benign or malignant ovarian tumors (n=111 and n=37, respectively). They found that the positivity rate of sB7-H4 was significantly higher in patients with malignant OC compared to those with benign ovarian lesions, suggesting that B7-H4 may serve as a potential adjunctive plasma biomarker (49). The meta-analysis conducted by Zhu et al. indicated that overall diagnostic sensitivity and specificity of serum B7-H4 in OC were 0.782 (95% confidence interval [CI]: 0.732–0.825) and 0.870 (95% CI: 0.804–0.916), respectively. The Summary Receiver Operating Characteristic Curve(SROC) analysis revealed that the combined detection of B7-H4 and CA-125 for OC had a higher Area Under Curve(AUC) than B7-H4 alone (0.94 vs. 0.86) (50). The combination of B7-H4 and CA-125 may enhance the early and effective screening of OC. However, further validation through additional multicenter and randomized controlled trials is needed to more precisely determine the clinical utility of B7-H4 (19, 44, 45, 51, 52). Mach et al. detected soluble B7-H4 (sB7-H4) in 12 out of 85 patients with advanced epithelial ovarian cancer (EOC) and also collected circulating tumor cells (CTCs). They indicated that positivity for sB7-H4 in EOC patients is associated with poorer overall survival (OS) and platinum resistance (53).

The majority of studies have linked B7-H4 with unfavorable prognostic outcomes and a heightened recurrence rate in OC (22, 23, 54). However, Liang et al. demonstrated that heightened B7-H4 expression in tumor tissue of patients with ovarian plasmacytoid carcinoma does not correlate with OS or disease-free survival, but it is associated with advanced tumor stage (43). Overall, confounding factors such as different B7-H4 assays and sample sizes may contribute to different results. Further investigations are warranted to elucidate the expression profiles of B7-H4 across various pathological subtypes of OC and its correlation with tumor grading, staging, and prognosis.

In summary, B7-H4 emerges as a promising biomarker for early cancer detection, prediction of immunotherapy response, and evaluation of patient prognosis in OC (21, 23, 36–38, 53, 55). Further exploration is warranted into the regulatory mechanisms driving B7-H4 overexpression in ovarian carcinogenesis, along with delineating the specific impacts of its various subcellular localizations within tumor cells, particularly the role of intracellularly expressed B7-H4, which necessitates deeper investigation.

## 4 The role of B7-H4 in tumor immunity of OC

### 4.1 Immune regulation by B7-H4 in the TME of OC

Tolerance in the TME is a dynamic and intricate process characterized by a network of interactions among diverse cell types (56). The immunosuppressive TME of OC predominantly comprises T and B lymphocytes, T regs, NK cells, tumor-associated macrophages (TAMs), and myeloid-derived suppressor cells (MDSCs). Antigen-presenting cells (APCs) play a pivotal role in initiating and sustaining tumor-associated antigen (TAA)-specific T-cell immunity, which significantly impacts survival and recurrence rates in OC (31, 57). TAMs, the most abundant APCs within the OC TME, prominently express B7-H4, with levels surpassing 70% (52). The intensity of B7-H4 expression with TAM in OC positively correlates with the intratumoral Treg population. Moreover, the presence of more Tregs further releases more IL-6 and IL-10, which continue to induce B7-H4 expression on APCs, including TAM and M2 macrophages (27, 31, 34, 52, 58, 59). This cascade establishes a positive feedback loop that fosters an immunosuppressive TME. The interaction between B7-H4 and its receptor on T cells leads to diminished T cell proliferation by limiting the entry of CD4+ and CD8+ T cells into the cell cycle and decreasing their division rate. Furthermore, it inhibits the secretion of cytokines by T cells, such as IL-2, and IFN-γ (24, 25, 30, 60). Additionally, B7-H4 overexpression can protect tumor cell from apoptosis and facilitates their growth in OC (19). Cai et al. found that B7-H4 expression on APC negatively correlates with infiltration and cytolytic function of CD8+ TILs, but no significant correlation was found between B7-H4 expression and tumor grade or stage (40). These observations may partly stem from incongruent findings attributable to variances in B7-H4 assay methodologies and the limited scale of sampled populations.

Some studies have presented alternative perspectives. An investigation focusing on ovarian serous carcinoma indicated a positive correlation between B7-H4 expression and TILs, while noting that B7-H4 expression was not inducible by interleukin-4 (IL-4), IL-6, or IL-10. This suggests that B7-H4 might not actively participate in immune evasion mechanisms, although it does affirm that heightened B7-H4 expression correlates with higher tumor grade and lower overall survival (61). Pagnotti G M et al. found no association between B7-H4 protein level and the infiltration degree of CD3+, CD4+, CD8+, and CD14+ lymphocytes in serous or endometrioid OC, but observed an inverse correlation in clear cell OC (62). MacGregor et al. reported no discernible relationship between B7-H4 expression levels and the abundance or phenotype of T and B cells, nor any interaction between B7-H4 and other inhibitory ligands such as PD-1, Tim-3, or LAG3 (26).

**Figure 1:**
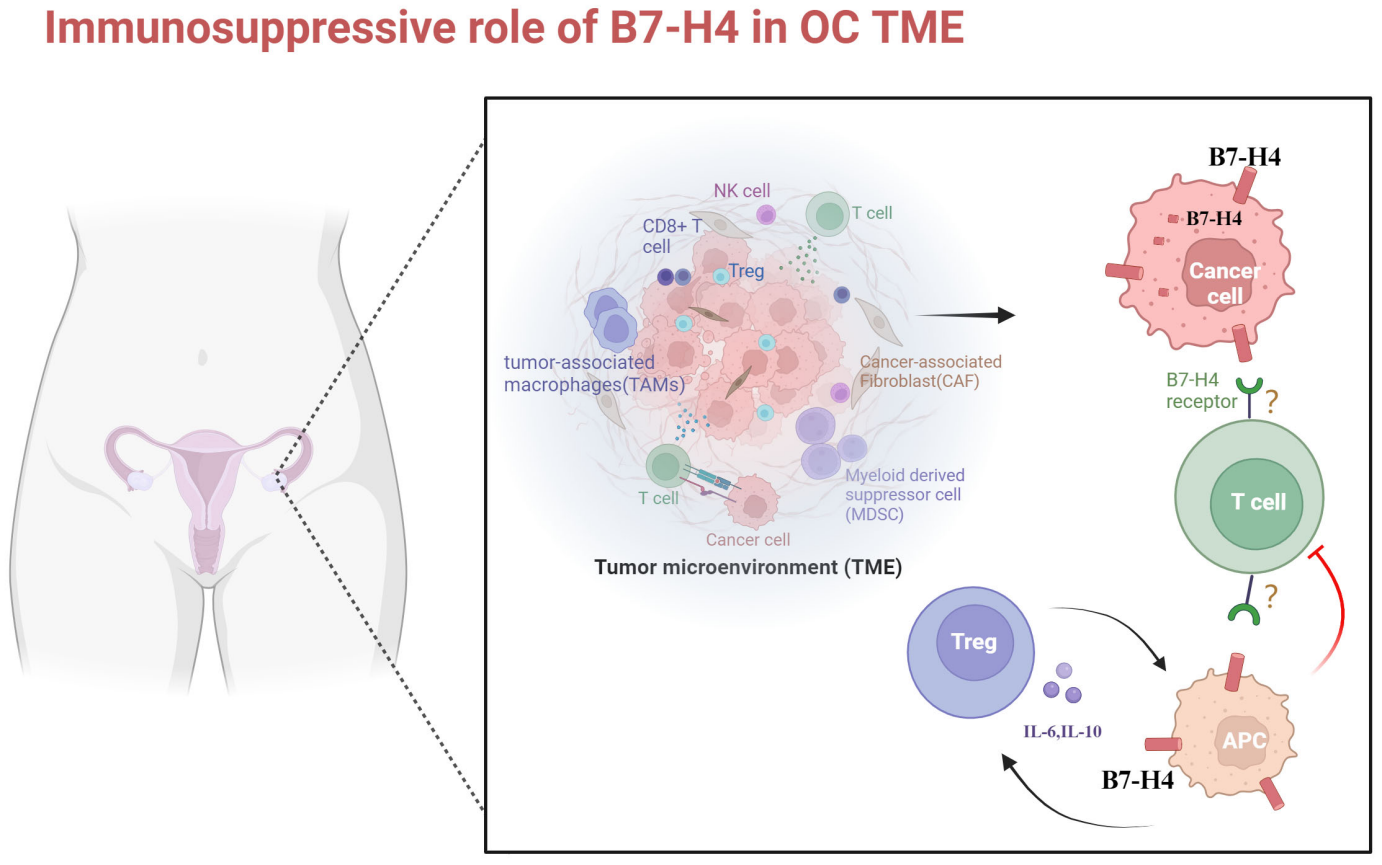
The role of B7-H4 in microenvironmental immunosuppression in ovarian cancer. OC is a cold tumor. The immunosuppressive TME of OC predominantly comprises T and B lymphocytes, T regs, NK cells, TAMs, and MDSCs. B7-H4 is highly expressed in OC cells and APCs. Its elevated expression in OC cells inhibits apoptosis. Upon binding to unidentified receptors on T cells, B7-H4 inhibits T cell proliferation and cytokine secretion. The expression intensity of B7-H4 on APCs positively correlates with the number of Tregs, leading to increased release of IL-6 and IL-10. This further induces overexpression of B7-H4 on APCs, fostering an immunosuppressive TME in OC. TME, tumor microenvironment; NK cell, natural killer cell; TAMs, tumor-associated macrophages; MDSCs, myeloid-derived suppressor cells; APC, antigen-presenting cell. Created with BioRender.com.

It has been reported in other tumors that B7-H4 participates in modulating intracellular oncogenic signaling pathways, with intracellular localization of B7-H4 facilitating signals conducive to tumor cell proliferation. As mentioned before, a high intracellular B7-H4 expression state has been found in OC tumor cells. Difference in subcellular localization of B7-H4 suggests potential differential roles in tumor development, a facet yet to be comprehensively elucidated in the context of OC. In conclusion, B7-H4 in OC TME inhibits the activation and function of effector T cells, promotes the suppression of immune responses by immunosuppressive cells, and protects tumor cells from apoptosis. These contributes to tumor immune evasion and tumor progression (**Figure 1**).

### 4.2 B7-H4 is involved in the antitumor immunity of OC and interacts synergistically with other immune checkpoints

In recent years, it has emerged that heightened expression of B7-H4 within tumor-infiltrating antigen-presenting cells in the microenvironment of hepatocellular carcinoma suppresses the proliferation and cytotoxicity of CD8+ TIL cells by inducing up-regulation of the transcription factor eomesodermin. This phenomenon promotes TIL depletion, thereby facilitating tumor progression and inhibiting antitumor immune responses (27). They also revealed that an intricate interplay exists between B7-H4 signaling and other pivotal pathways, such as immunomodulatory signaling. B7-H4 knockout notably impacts cell cycle and NF-kB pathways, resulting in up-regulation of co-stimulatory ligands or receptors (e.g., ICOSL, CD27, CD28), while concurrently down-regulating certain co-inhibitory receptors (e.g., LAG3, CTLA4) and up-regulating others (e.g., LILRB4, PDCD1).

However, in OC, the co-expression of B7-H4 with other immune checkpoints and the specific mechanisms of their interactions have not yet been fully elucidated. Cai et al. collected fresh tumor tissue from 32 chemotherapy-naive patients with newly diagnosed OC and found a notable positive correlation between the expression of B7-H4 and several immune co-inhibitory checkpoints, including CTLA4, HAVR2, LAG3, TIGIT, and C10orf54 (40).

Previous researches have indicated a negative or non- correlation between B7-H4 and PD-L1 in various tumors, characterized by low rates of double-positive expression (63–67). The same expression profile has been observed in OC. However, elevated levels of co-expression of B7-H4 and PD-L1 have been found in ovarian clear cell carcinoma, suggesting a potential suitability for combinatorial therapeutic approaches (62, 68), although further validation through trials is needed.

Cai et al. also demonstrated that B7-H4 was mainly involved in diverse OC anti-tumor immune responses and signaling pathways, including but not limited to IL-2/signal transducer and activator of transcription (STAT)5 signaling, the p53 pathway, mammalian target of rapamycin complex 1 (mTORC1) signaling, and apoptosis (40). Although the specific mechanisms remain incompletely understood, the important role of B7-H4 in antitumor immunity in OC has been emphasized, suggesting that it is a potential target for immunotherapy and combinatorial immunotherapy.

## 5 Preclinical or clinical trials of B7-H4 in immunotherapy of OC

Currently, the focus of immunotherapies targeting B7-H4 in OC revolves around ICI, adoptive cell therapy(ACT), and antibody-drug conjugates (ADC) (69). However, the efficacy of ICI monotherapy in OC is suboptimal, necessitating further investigation into novel immune targets and combination therapeutic modalities, which are still in the exploratory and clinical trial phases and require additional validation through rigorous research. B7-H4 presents an attractive target for cancer immunotherapy, with potential blockade via various mechanisms, including monoclonal antibodies (mAbs), single chain fragment variables(scFv), antibody-drug conjugates (ADCs), CD3 bispecific antibodies (BiTEs), and chimeric antigen receptor T cells (CAR-Ts) in OC (70–73). We provide a comprehensive summary of both preclinical and clinical trials investigating B7-H4 in the context of OC immunotherapy in recent years (**Tables 1**, **2**).

**Table 1:** Preclinical trials of B7-H4 in immunotherapy for ovarian cancer (PDX, patient-derived xenograft; CDX: cell line-derived xenograft; ADCC, antibody-dependent cellular cytotoxicity; ADCP, Antibody dependent cell-mediated phagocytosis).

Experimental Materials	Intervention	Mechanisms	References
OC cell line: SKOV3	Drug: CH17 Type: neutralizing antibody	(1) Enhances the activation and proliferation of CD8+ T cells (2) Induces ADCC and dose-dependent cytotoxicity (3) Exhibits synergistic effects when used in combination with PD-1 antibodies	(74)
Fresh primary OC cells Mice bearing OC PDX models	Drug and type: monoclonal antibody	(1) Restores tumor antigen-specific T-cell activation and inhibits tumor progression by reversing T-cell immunosuppression (2) Enhances ADCC effects (3) Reduces the concentrations of VEGF and TGF-β within the tumor microenvironment	(42, 70)
Mice bearing OC PDX models	Drug: ABL 103 Type: bispecific antibody	(1) Blocks B7-H4-mediated T cell inhibition (2) Effectively inhibits tumor progression in a dose-dependent manner. (3) Establishes long-term antitumor memory responses	(75)
OC cell line: OVCAR3 Mice bearing OC PDX models	Drug and type: CAR T cell therapy	(1) Induces dose-dependent secretion of IFN-γ, IL-2, TNF-α, and MIP-1α (2) Enhances the cytolytic activity against OVCAR3 cells. (3) Long-term engraftment of B7-H4 CAR T cells mediates lethal, off-tumor toxicity	(71)
Thirteen ovarian cancer cell lines, including OAW28, PEO1, PEO2, and others Mice bearing OC PDX models	Drug and type: ADC	(1) Exhibits target-specific growth inhibition of OC cell lines (2) significantly decreases cell viability and colony formation (3) Induces concentration-dependent cell-cycle arrest and DNA damage, ultimately leading to apoptosis (4) A single dose of B7-H4-ADC results in sustained tumor regression and increased survival (5) Mediates ADCC	(47)
Mice bearing OC PDX models	Drug: XMT-1660 Type: ADC	Demonstrates potent antitumor activity, leading to complete tumor regression	(92)
Mice bearing OC PDX models	Drug: SGN-B7H4V Type: ADC	(1) Eliminates tumor cells through direct cytotoxicity (2) Mediates ADCC and ADCP	(93)

**Table 2:** Clinical trial of immunotherapy targeting B7-H4 in ovarian cancer (NA = not applicable).

Cancer Type	Intervention	Phase	Dates	Clinical trial identified/Ref
Ovarian Cancer Fallopian Tube Cancer Primary Peritoneal Cancer Endometrial Cancer	Drug: HS-20089 Type: ADC	II	Study Start: 2023-12-31 Study Completion: 2027-12-31	NCT06014190
Ovarian Neoplasms Fallopian Tube Neoplasms Peritoneal Neoplasms Other solid tumors	Drug: SGN-B7H4V Type: ADC	I	Study Start: 2022-01-12 Study Completion: 2027-01-31	NCT05194072
Ovarian Cancer Other solid tumors	Drug: AZD8205 Type: ADC	I II	Study Start:2021-10-18 Study Completion: 2025-06-30	NCT05123482
Ovarian Cancer Other solid tumors	Drug: XMT-1660 Type: ADC	I	Study Start: 2022-08-15 Study Completion: 2027-05	NCT05377996
Ovarian Cancer Other solid tumors	Drug: GEN1047 Type: Bispecific antibody	I II	Study Start: 2021-12-13, 2021 Study Completion: 2026-06-30	NCT05180474
Ovarian Cancer Other advanced or metastatic solid tumors	Drug: ABL103 Type: Bispecific antibody	I	Study Start: 2023-11-07 Study Completion: 2027-11-15	NCT06126666
Ovarian Cancer Other advanced or metastatic solid tumors	Drug: NC762 Type: Monoclonal antibody	I II	Study Start: 2021-06-30 Study Completion: 2024-10	NCT04875806
Ovarian Cancer Other advanced solid tumors	Drug: FPA150 Type: Monoclonal antibody	I	Study Start: 2018-03-27 Study Completion: 2021-05-10 Results Submitted: 2024-03-14, yet NA	NCT03514121

### 5.1 Preclinical studies of B7-H4 in OC

#### 5.1.1 B7-H4 and ICI

Enhancing the tumor-killing capability of immune cells by reinstating T cell functionality through the blockade of B7-H4 inhibitory immune checkpoint signaling is a very competitive option. Miao et al, developed a neutralizing antibody against B7-H4 named CH17, can enhance antigen specific T cell responses in OC cell line (SKOV3). Notably, the B7-H4 antibody(CH17) exhibited robust *in vivo* anti-tumor efficacy in a human T-cell transplantation xenograft model and synergistic effects when combined with anti-PD-1 antibody (74). Monoclonal antibody directed against B7-H4 have shown promising outcomes in inhibits tumor progression by blocking T-cell immunosuppression and augmenting antibody-dependent cellular cytotoxicity(ADCC) effects in OC, concurrently reducing the concentrations of VEGF and TGF-β within the TME (42, 70).

On the other hand, development of drugs featuring dual inhibitory action represents a particularly promising avenue. In recent research, ABL103, a novel T-cell engaging bispecific antibody designed to target both B7-H4 and 4-1BB, was developed. ABL103 operates through a dual mechanism, enhancing T cell functionality by inhibiting B7-H4 while simultaneously activating 4-1BB. This novel approach has yielded robust *in vitro* and vivo anti-tumor activity, coupled with a favorable safety profile, achieved through B7-H4-dependent 4-1BB activation in the TME of OC. Moreover, long-term anti-tumor response memory can be established in OC model mice (75). Chang et al, engineered a novel anti-B7-H4/IL15 fusion antibody, which enhances the immunogenicity of the TME by fostering the proliferation of CD8+ T cell and facilitating the elimination of B7H4-expressing tumor cells by activated immune cells, including ADCC dependent cellular cytotoxicity (76).These studies strongly suggest bispecific antibodies targeting B7-H4 is a promising therapeutic agent.

Of note, it is possible that the combination of PD-1 and B7-H4 blockade may offer enhanced efficacy and safety compared to the combination of PD-1 and CTLA-4 blockade. There exists the potential for synergistic blockade by concurrently targeting B7-H4 and PD-1 in OC, though this necessitates further validation. In addition, the safety and clinical efficacy of B7-H4 in combination with other ICIs in OC need to be further explored. In conclusion, given OC’s potential for mediating immunosuppression through multiple immunosuppressive checkpoints, and the likelihood of complementary effects among different inhibitory checkpoints, monotherapy with a single immune checkpoint inhibitor is expected to be insufficient. Therefore, combination therapy with ICIs is imperative in the future, especially in the treatment of advanced or recurrent OC, emphasizing the importance of combination drug regimens.

#### 5.1.2 B7-H4 and chimeric antigen receptor T cells

Chimeric antigen receptors (CARs) are synthetic receptors engineered to endow T cells with the capability to recognize tumor-associated antigens (TAAs) in a manner independent of major histocompatibility complex (MHC) presentation (77–80). CAR-T therapy stands as the pioneering genetically modified cell-based therapeutic endorsed by the US Food and Drug Administration (81–84). However, obstacles to the use of CAR-T in solid tumors such as OC, primarily attributed to the heterogeneity of TAA, limited transport and infiltration within solid tumors owing to their substantial dimensions, and the ubiquitous expression of target antigens across vital healthy tissues.

The expression of B7-H4 on tumor cells surfaces provides an avenue for targeted therapy utilizing T cells expressing CARs. Notably, in a mouse ovarian tumor xenograft model, a CAR targeting both human and mouse B7-H4 exhibited efficacy in inducing tumor regression, marking the inaugural instance of CAR T cell therapy targeting B7-H4. However, this therapeutic approach precipitated delayed and ultimately fatal lung tumor toxicity after 6-8 weeks, thereby constraining further applications of CAR-T (71). Analysis of post-mortem mice revealed that multi-organ lymphocytic infiltration was predominantly associated with membranous B7-H4-positive tissue expression, with extensive histological lesions observed in associated with B7-H4(+) expression. Notably, lesions were also evident in tissues lacking B7-H4 expression. Furthermore, while robust expression of B7-H4 protein was observed in ovarian cancer tissues, varying degrees of expression, ranging from weak to strong, were also detected in human mammary glands, kidneys, pancreatic islet cells, esophagus, salivary glands, and liver, thus updating prior assumptions regarding the spectrum of B7-H4 expression in healthy human tissues. Consequently, the clinical application of CAR-T targeting B7-H4 in OC warrants careful consideration, particularly concerning the potential for fatal non-tumor toxicity associated with prolonged use.

Efforts to enhance the efficacy of B7-H4 CAR-T therapy include strategies for reducing dosing duration, such as utilizing transient CAR expression through inducible suicide genes (85) or RNA electroporation (77, 86), as well as investigating combination therapies to augment anti-tumor efficacy. Additionally, exploring the withdrawal of B7-H4 CAR-T prior to onset of lethal toxicity represents a prospective approach. Despite the potential for a broad spectrum of toxic effects, the excellent targeting effect and precise anti-tumor efficacy of B7-H4 CAR-T still underscore its status as a promising therapeutic avenue in OC, deserving more research in the future.

#### 5.1.3 B7-H4 and ADCs

B7-H4 presents a viable target for cytotoxic therapy or immune-mediated killing (87). Monoclonal antibodies against B7-H4 can induce cell killing through ADCC, as previously mentioned (42). However, a more direct cytotoxic strategy involves the use of antibody-drug conjugates (ADCs) (88), a well-established class of targeted therapies across various cancers, including OC (89). Following recent FDA approvals of mirvetuximab soravtansinegynx(Elahere) for OC, ADCs, often regarded as “smart chemotherapy”, deserve more investment and greater application prospects (90, 91). ADCs comprise an antibody, a cytotoxic payload, and a linker connecting them. The tumor-specific antigenic properties of the B7-H4 protein, characterized by its heightened expression in malignant tumors while low or no expression in normal tissues, make it one of the promising targets for ADCs.

A recent study has yielded promising outcomes regarding ADC agents targeting B7-H4 in patient-derived xenograft (PDX) models of OC. Notably, in PDX models of PARPi and platinum-resistant HGSOC, scheduled administration of B7-H4-ADC demonstrated sustained tumor regression and increased overall survival (OS) (47). Moreover, this study elucidated that B7-H4-ADC induces concentration-dependent cell-cycle arrest and DNA damage. In addition, B7-H4-ADC has bystander killing activity, thereby augmenting its efficacy and potentially targeting OC cases with low or moderate B7-H4 expression levels. Toader et al, reported XMT-1660, an ADC targeting B7-H4, showing potent anti-tumor activity in a PDX model of OC. It revealed ADCs may be effective in patients refractory or resistant to immune checkpoint inhibitors (92). Gray et al, have pioneered a novel investigational vedotin ADC named SGN-B7H4V. They validated its potent antitumor activity in the OC PDX model and observed enhanced effectiveness when combined with anti-PD-1 agents (93). Kinneer et al, developed AZD8205, a B7-H4-directed ADC, and preliminary data indicate that its combination with PARPi can sensitize triple negative breast cancer(TNBC) PDX tumors expressing low levels of B7-H4 (39).

**Figure 2:**
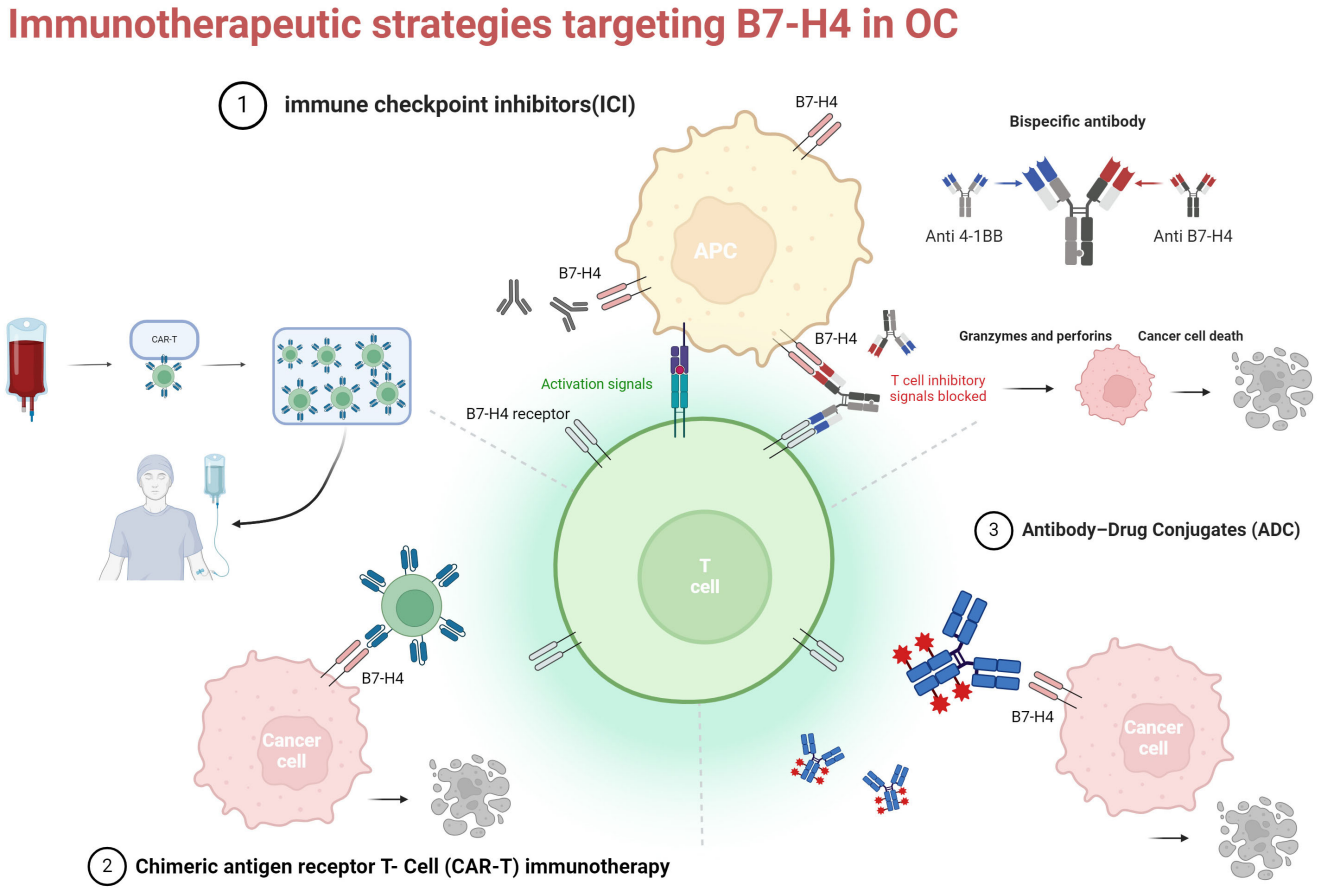
Three current immunotherapy strategies for targeting B7-H4 in OC. ICI, immune checkpoint inhibitors: monoclonal antibodies and bispecific antibodies (example illustration with bispecific antibodies targeting B7-H4 and 4-1BB); CAR-T, chimeric antigen receptor t-cell; ADC, antibody-drug conjugates. Created with BioRender.com.

Overall, the current studies reinforce the notion of B7-H4 as an attractive target for ADCs, indicating potential for enhanced efficacy when utilized in combination with immunosuppressants or PARP inhibitors (**Figure 2**).

### 5.2 Clinical trials of B7-H4 in OCA

Phase 1a/1b clinical trial evaluating the anti-B7-H4 antibody(FPA150) commenced in patients with advanced solid tumors, including OC, aiming to assess the safety, tolerability, and preliminary efficacy of FPA150 either as monotherapy or in combination with anti-PD1 therapy (38, 94). Initial findings reported in 2019 showed a favorable safety profile for FPA150; however, subsequent data regarding its efficacy as monotherapy in OC patients have not been disclosed. The clinical Phase I and Phase II trials of the monoclonal antibody NC762 and the bispecific antibody GEN1047 are currently underway. Another Phase I clinical trial of the bispecific antibody ABL-103 is also currently underway.

Several B7-H4-ADCs are being explored in patients afflicted with metastatic or recurrent OC. The clinical trials of SGN-B7H4V, AZD8205 and XMT-1660 in Phase I or Phase II hold promise, and we eagerly await forthcoming data to clarify their efficacy in OC. Recently, initial results have been published from a Phase I investigation of HS-20089, a novel B7-H4-targeted ADC. HS-20089 showed great anti-tumor activity in OC, yielding an objective response rate (ORR) of 66.7% and a disease control rate (DCR) of 100% in platinum-resistant ovarian cancer(PROC) (95). Subsequent research data are eagerly anticipated (**Table 1**).

## 6 Conclusion

We summarize the expression and clinical significance of B7-H4 in OC, discuss the current landscape of immunotherapy research in OC, recent advancements, and delineate future research directions aimed at deeper elucidation of B7-H4’s role in OC. The expression pattern of B7-H4 distinguishes it from PD-1 and CTLA-4, with high mRNA expression and low protein expression in normal tissues, while demonstrating widespread expression in malignant tumors, particularly in OC. This distinct expression pattern suggests that B7-H4 holds greater tumor specificity and sensitivity compared to PD-1 and CTLA-4, rendering it a promising emerging target for tumor therapy. Compared with the studies of B7-H4 in other types of tumors, there are still a lot of mist unknown in OC, underscoring the imperative for in-depth exploration of B7-H4 in the context of OC.

Despite the fact that much remains unknown about B7-H4 in OC, evidence has already substantiated its status as a highly promising and emerging immunotherapeutic target for OC. In summary, it is imperative to elucidate the immunoregulatory pathways and expression patterns of B7-H4 in ovarian cancer, identify its receptor(s), investigate downstream mechanisms of B7-H4 with effector T cells and other APC surface receptors, examine its role within the ovarian cancer microenvironment, including potential variances across different histological subtypes. Explore predictive biomarkers for B7-H4 immunotherapy specificity, mechanisms of drug resistance, devise combination therapies with different immune checkpoints, and develop multi-strategy immunotherapeutic drugs targeting B7-H4. These endeavors hold the promise of expanding the repertoire of immunotherapeutic options and improving the prognosis of OC patients.
